# HIV-1 Inhibits Autophagy in Bystander Macrophage/Monocytic Cells through Src-Akt and STAT3

**DOI:** 10.1371/journal.pone.0011733

**Published:** 2010-07-22

**Authors:** Jennifer Van Grol, Cecilia Subauste, Rosa M. Andrade, Koh Fujinaga, Julie Nelson, Carlos S. Subauste

**Affiliations:** 1 Department of Pathology, Case Western Reserve University School of Medicine, Cleveland, Ohio, United States of America; 2 Division of Infectious Diseases and HIV Medicine, Department of Medicine, Case Western Reserve University School of Medicine, Cleveland, Ohio, United States of America; 3 Department of Ophthalmology and Visual Sciences, Case Western Reserve University School of Medicine, Cleveland, Ohio, United States of America; 4 McDermott Center for Human Growth and Development, University of Texas Southwestern Medical Center, Dallas, Texas, United States of America; 5 Department of Medicine, University of Cincinnati College of Medicine, Cincinnati, Ohio, United States of America; 6 Center for AIDS Research, University of North Carolina, Chapel Hill, North Carolina, United States of America; Tsinghua University, China

## Abstract

Autophagy is a homeostatic mechanism of lysosomal degradation. Defective autophagy has been linked to various disorders such as impaired control of pathogens and neurodegeneration. Autophagy is regulated by a complex array of signaling pathways that act upstream of autophagy proteins. Little is known about the role of altered regulatory signaling in disorders associated with defective autophagy. In particular, it is not known if pathogens inhibit autophagy by modulation of upstream regulatory pathways. Cells infected with HIV-1 blocked rapamycin-induced autophagy and CD40-induced autophagic killing of *Toxoplasma gondii* in bystander (non-HIV-1 infected) macrophage/monocytic cells. Blockade of autophagy was dependent on Src-Akt and STAT3 triggered by HIV-1 Tat and IL-10. Neutralization of the upstream receptors VEGFR, β-integrin or CXCR4, as well as of HIV-1 Tat or IL-10 restored autophagy in macrophage/monocytic cells exposed to HIV-1-infected cells. Defective autophagic killing of *T. gondii* was detected in monocyte-derived macrophages from a subset of HIV-1^+^ patients. This defect was also reverted by neutralization of Tat or IL-10. These studies revealed that a pathogen can impair autophagy in non-infected cells by activating counter-regulatory pathways. The fact that pharmacologic manipulation of cell signaling restored autophagy in cells exposed to HIV-1-infected cells raises the possibility of therapeutic manipulation of cell signaling to restore autophagy in HIV-1 infection.

## Introduction

Autophagy is a conserved degradative process whereby a double membrane vesicle, an autophagosome, forms around a portion of the cytoplasm or an organelle and fuses with a late endosome/lysosome resulting in enzymatic degradation of its cargo [1], [2]. Autophagy was originally described as a homeostatic mechanism that provides energy and substrates for synthesis of essential macromolecules during starvation [1], [2]. However, it is now recognized that autophagy is involved in many other processes. Indeed, deficiency in autophagy promotes disorders such as cancer, neurodegeneration, myopathy, senescence and susceptibility to infections [3].

Autophagy can act as an innate anti-microbial mechanism against several pathogens both *in vitro* [4], [5], [6] and *in vivo* [7], [8], [9], [10]. Autophagy is also an important component of adaptive immunity against pathogens. IFN-γ or CD40 enables macrophages to kill *Mycobacterium tuberculosis* or *Toxoplasma gondii* via autophagy, respectively [4], [11], [12]. In addition, autophagy has been reported to enhance antigen presentation [13], [14]. In a model of immunization with *M. tuberculosis*-infected dendritic cells, *in vitro* treatment of these cells with a pharmacologic stimulator of autophagy improved the efficacy of immunization [15].

Autophagy-related (Atg) proteins mediate the formation of autophagosomes. In contrast to the highly conserved nature of Atg proteins, the regulatory molecules that control autophagy are varied, including JNK, ERK1/2, AMP kinase, class I and class III PI3K, Akt, mTOR, JAK, STAT, eIF2α kinases, DAPK, Bcl-2 family proteins, the p53 tumor suppressor, ubiquitination, the ER-membrane-associated protein, Ire-1 [16]. Little is known about the role of these signaling pathways in diseases linked to dysregulation of autophagy.

Questions that are receiving increased attention are whether and how pathogens inhibit autophagy. Pathogens have been reported to inhibit autophagy by expressing virulence factors that impair the function of Atg proteins [6], [8], [17]. However, to our knowledge, there is no demonstration of whether pathogens interfere with autophagy by modulating upstream signaling mechanisms. Addressing this question is key to attempts to modulate autophagy for therapeutic purposes. While approaches that target Atg proteins are likely to result in generalized, non-specific modulation of autophagy, approaches aimed at preventing dysregulation of the pathways upstream of autophagy are more likely to be of therapeutic benefit.

It has recently been reported that HIV-1 interacts with the autophagy pathway in cells infected with the virus. Autophagy is stimulated in macrophages infected with HIV-1 [17], [18]. However, the degradative stage of autophagy is prevented by the interaction between viral protein Nef and the autophagy protein Beclin 1 [17]. One of the important aspects of the pathophysiology of HIV-1 infection is that despite the fact that most cells of the immune system are not infected with the virus, HIV-1 can affect the function of non-infected cells creating a more global effect to HIV-1 infection. In this regard, cell-free supernatants from microglia obtained from SIV-1-infected monkeys inhibit autophagy in bystander neurons [19]. However, it was not determined if the inhibition of autophagy is a direct consequence of SIV-1. Thus, it is not known if HIV-1 inhibits autophagy in bystander cells, and if this is the case, by what mechanisms such an inhibition occurs. We uncovered that HIV-1 inhibits autophagy in bystander macrophage/monocytic cells by acting through counter-regulators of this process, Src, Akt, and STAT3. This is the first evidence of a pathogen targeting signals upstream of autophagy to impair this process. These findings are likely relevant to key aspects of HIV-1-induced disease since they may contribute to susceptibility to infections and immune evasion by inhibition of antigen recognition as well as neuro-degeneration associated with HIV-1. Importantly, we also report that it is possible to prevent HIV-1 from inhibiting autophagy by pharmacologic manipulation of cell signaling.

## Results

### HIV-1 blocks autophagic killing of *T. gondii* in bystander macrophages

**Figure 1:**
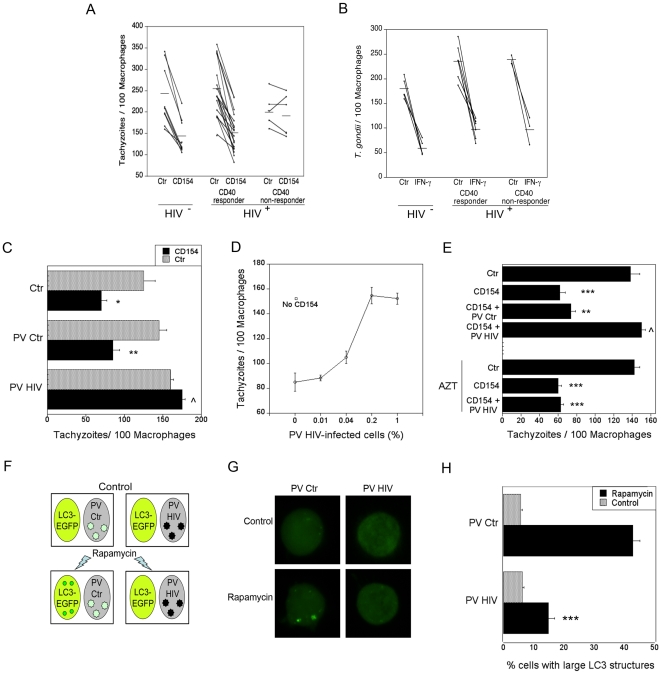
HIV-1 inhibits autophagy in bystander macrophages/monocytic cells. *A* and *B*, MDM from healthy controls and HIV-1^+^ patients were incubated with or without CD154 (3 µg/ml) (*A*) or IFN-γ (200 U/ml) (*B*) followed by challenge with *T. gondii* tachyzoites. The number of parasites per 100 macrophages was assessed by light microscopy 24 h post-challenge. HIV-1^+^ patients were classified as non-responder when their macrophages exhibited a percentage decrease in parasite load that was less than the 10^th^ percentile of the percentage decrease in parasite load observed in macrophages from healthy controls. *C*–*E*, MDM from healthy controls were infected with pseudotyped HIV-1 (PV HIV) or pseudotyped control virus (PV Ctr) and were incubated with a monolayer of uninfected macrophages. In certain experiments macrophages were treated with or without zidovudine (AZT) 2 h after incubation with pseudotyped HIV-1 (*E*). Macrophage monolayers were treated with or without CD154 followed by challenge with *T. gondii* and assessment of parasite load at 24 h. *F*, Schematic representation of MonoMac6 cells infected with pseudotyped HIV-1 (PV HIV) or pseudotyped control virus (PV Ctr) incubated with uninfected MonoMac6 cells transfected with LC3-eGFP. Cells were treated with or without rapamycin (1 µM) and assessed for autophagy by expression of large (≥1 µm) LC3^+^ structures. *G*, Flourescent images of LC3-eGFP^+^ cells as treated in panel (F). *H*, Quantification of autophagic cells as treated in panel (F). Data are representative of 4 independent experiments presented as means ± SEM; ***p≤0.001; **p≤0.01; *≤0.05; ∧p≥0.05.

CD40 stimulation of macrophages results in killing of *T. gondii* through autophagy [11], [12]. To determine whether autophagy is affected by HIV-1, we examined autophagic killing of *T. gondii* in macrophages from HIV-1^+^ patients. Monocyte-derived macrophages (MDM) from HIV-1^+^ patients and healthy controls were incubated with or without CD154 (CD40 ligand) followed by challenge with *T. gondii*. MDM from 5 of a total of 23 HIV-1^+^ patients exhibited defective induction of anti-*T. gondii* activity (Figure 1A). Impaired anti-*T. gondii* activity was reproducible since similar results were obtained when MDM were retested 2 to 3 different times. The group of HIV-1^+^ patients with defective CD40-induced toxoplasmacidal response will be referred to as CD40 non-responders. CD40-induced anti-*T. gondii* activity did not correlate with CD4 counts or plasma viral load (R = 0.02; p = 0.76; data not shown). Impaired CD40-dependent toxoplasmacidal activity was not due to differences in CD40 expression on MDM (CD40 corrected mean fluorescence intensity: controls 26.6±4.4; CD40 responders 29.9±4.5; CD40 non-responders 32.6±6.4; p>0.1). While CD40 induces *T. gondii* killing through autophagy, IFN-γ works independently of autophagy to kill *T. gondii* [11]. Indeed, in marked contrast to results obtained with CD40-stimulated MDM, anti-*T. gondii* activity induced by IFN-γ was similar in all groups (Figure 1B).

Replication-competent virus can be present in a small percentage of peripheral blood monocytes from HIV-1^+^ patients, even in those receiving anti-retroviral therapy and with undetectable plasma viral load [20]. This raised the possibility that MDM productively infected with HIV-1 may impair CD40-dependent autophagic killing of *T. gondii* in MDM not infected with HIV-1. To address this possibility, MDM from healthy controls were infected with *env*-deficient, *nef*-deficient Heat Stable Antigen (HSA)-encoding HIV-1. The virus was pseudotyped with VSV-G envelope to enable initial infection. This pseudotyped HIV-1 is competent for a single round of replication since it lacks expression of gp160. Expression of HSA or intracellular p24 was used to quantitate the percentage of macrophages infected with pseudotyped HIV-1. Uninfected MDM incubated with pseudotyped HIV-1-infected MDM were unable to acquire anti-*T. gondii* activity in response to CD40 stimulation (Figure 1C). This inhibitory effect took place even when a low percentage of the total MDM population was infected with HIV-1 (Figure 1D). To determine if the inhibition of anti-microbial activity required the expression of HIV-1 genes we treated cultures with the nucleoside analog reverse transcriptase inhibitor, zidovudine. Addition of zidovudine (AZT) 2 h after incubation with HIV-1, prevented expression of HIV-1 genes in MDM as assessed by intracellular p24 (not shown). While HIV-1-infected MDM incubated in medium alone inhibited CD40-dependent anti-*T. gondii* activity in non-HIV-1 infected MDM, zidovudine-treated HIV-1-infected MDM were unable to affect induction of anti-microbial activity (Figure 1E). Taken together, HIV-1 impairs CD40-dependent autophagic killing of *T. gondii* in primary macrophages not infected with HIV-1.

### Monocytic cells infected with HIV-1 block rapamycin-induced autophagy in bystander cells

We further determined whether HIV-1 impairs autophagy in bystander monocytic cells. Autophagy was assessed by examining the distribution of the autophagy protein LC3. MonoMac6 cells (a human monocytic cell line) transfected with LC3-eGFP were incubated with cells infected with pseudotyped HIV-1 or pseudotyped control virus and treated with or with out rapamycin, a stimulator of autophagy, and examined for the development of autophagosomes in LC3-eGFP^+^ cells using fluorescent microscopy (Figure 1F). MonoMac6 cells incubated with cells infected with pseudotyped control virus exhibited large LC3^+^ structures after rapamycin treatment (Figure 1G). In contrast, incubation with MonoMac6 cells infected with pseudotyped HIV-1 resulted in reduced presence of autophagosomes in bystander cells after rapamycin treatment as assessed by expression of large LC3^+^ structures (Figure 1G, 1H). A decrease in autophagosomes could be due to diminished autophagosome formation or increased degradation. MonoMac6 cells were treated with bafilomycin A_1_, an inhibitor of vacuolar ATPase, which prevents autophagosome degradation [21]. MonoMac6 cells infected with pseudotyped HIV-1 still inhibited the presence of autophagosomes in bystander cells when autophagosome degradation was prevented (Figure S1A). These results indicate that cells infected with HIV-1 inhibit autophagosome formation in non-infected monocytic cells.

Macrophages/monocytic cells exhibit enhanced autophagosome formation when they become infected with HIV-1 [17], [18]. Incubation of the human monocytic cell line THP-1 with T cells productively infected with HIV-1 results in HIV-1 infection of THP-1 cells and autophagosome formation [17], [18]. We used this model to examine whether autophagy was still inhibited in bystander monocytic cells when these cells were exposed to T cells that produce infectious HIV-1. THP-1 cells were incubated with pre-determined low concentrations of HIV-1-infected H9 T cells that did not increase autophagy in THP-1 cells. Addition of rapamycin caused a marked increase in autophagy in THP-1 cells if they were incubated with uninfected H9 cells but not with HIV-1-infected H9 T cells (Figure S1B). These results confirm that autophagy is inhibited in primary macrophages and monocytic cells exposed to cells infected with HIV-1.

### HIV-1 Tat inhibits induction of autophagy

**Figure 2:**
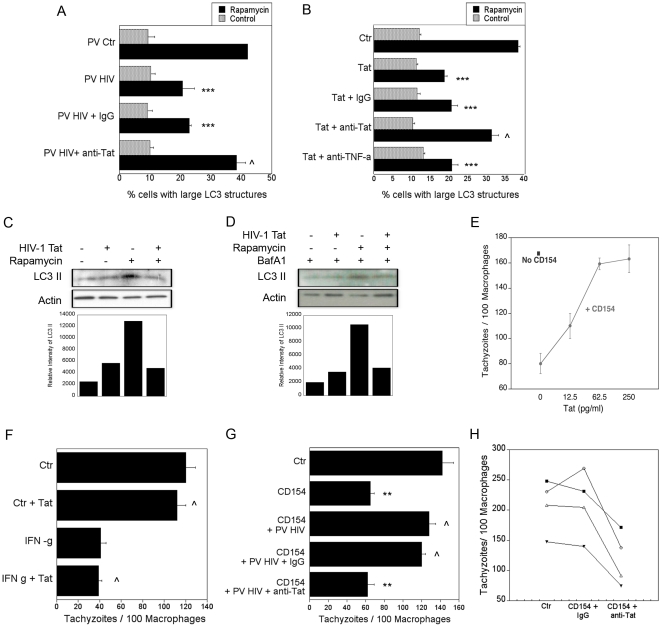
HIV-1 Tat inhibits autophagy. *A*, MonoMac6 cells infected with pseudotyped HIV-1 (PV HIV) or pseudotyped control virus (PV Ctr) were incubated with uninfected MonoMac6 cells transfected with LC3-eGFP overnight in the presence or absence of either a neutralizing anti-Tat or control IgG mAb. Cells were treated with or without rapamycin and assessed for autophagy by expression of large LC3^+^ structures. *B*, MonoMac6 cells transfected with LC3-eGFP were incubated overnight with HIV-1 Tat (100 pg/ml) plus either neutralizing anti-Tat, a neutralizing anti-TNF-α, or control IgG mAb. Cells were then stimulated with rapamycin. Autophagy was assessed by examining expression of large LC3^+^ structures. *C*, MDM were treated with or without HIV-1 Tat followed by rapamycin. Cell lysates were obtained at 2 h and used for immunoblot for LC3 II and actin. *D*, MDM were treated with or without HIV-1 Tat followed by rapamycin in the presence of bafilomycin A_1_ (Baf A1; 100 nM). Cell lysates were obtained at 2 h and used for immunoblot for LC3 II and actin. *E*, and *F*, MDM were treated with increasing concentrations of HIV-1 Tat (*E*) or HIV-1 Tat (60 pg/ml) plus IFN- γ (100 U/ml) (*F*) with or without CD154. Macrophages were challenged with tachyzoites of *T. gondii* and assessed for parasite load at 24 h. *G*, MDM infected with pseudotyped HIV-1 were incubated with uninfected macrophages. Cells were cultured in the presence of neutralizing anti-Tat or control mAb. After stimulation with CD154, monolayers were challenged with *T. gondii* and parasite load was assessed at 24 h. *H*, MDM from CD40 non-responder HIV-1^+^ patients were incubated with neutralizing anti-Tat or control mAb followed by stimulation with CD154. Macrophages were challenged with *T. gondii* and assessed for parasite load at 24 h. Each symbol represents a different patient. The number of parasites per 100 macrophages was examined at 24 h by light microscopy. Data are representative of 3 to 4 independent experiments presented as means ± SEM; ***p≤0.001, ** p≤0.01, ∧p≥0.05.

HIV-1 proteins can affect the function of cells not infected with the virus. Given that cells infected with *nef*-, *env*-deficient HIV-1 inhibited autophagy in bystander cells, we determined whether autophagy is inhibited by HIV-1 Tat. Tat is a cationic 86–101 amino acid polypeptide that acts as the main transactivator of HIV-1. HIV-1 Tat can be released from HIV-1-infected cells or dying cells [22], [23] and has been reported to exhibit pleiotropic extracellular effects on uninfected bystander cells [24], [25], [26], [27], [28], [29]. MonoMac6 cells infected with pseudotyped HIV-1 were no longer able to impair autophagy in LC3-eGPF^+^ bystander MonoMac6 cells in the presence of a neutralizing anti-Tat mAb (Figure 2A). Furthermore, incubation of MonoMac6 cells with a low concentration of HIV-1 Tat inhibited rapamycin-induced autophagy as assessed by the expression of large LC3^+^ structures (Figure 2B). The effect of HIV-1 Tat was specific since it was ablated by a neutralizing anti-Tat mAb (Figure 2B). In contrast, neutralization of TNF-α, an inducer of autophagy, did not restore autophagy in HIV-1 Tat treated macrophages (Figure 2B). The inhibition of autophagy by HIV-1 Tat was confirmed by examining the expression of LC3 isoforms. The amount of LC3 II directly correlates with the level of autophagy [2], [30], [31]. Immunoblot analysis of MDM treated with rapamycin revealed that the addition of HIV-1 Tat reduced LC3 II expression compared to controls (Figure 2C). HIV-1 Tat inhibited LC3 II expression even in the presence of bafilomycin A_1_ (Figure 2D). This supports previous experiments examining the presence of LC3 punctae in response to HIV-1 (Figure 1G, H; 2A, B), which indicate that HIV-1 inhibits the induction of autophagy in bystander cells.

Next, the effect of HIV-1 Tat on autophagic killing of *T. gondii* was examined. Figure 2E shows that low concentrations of HIV-1 Tat inhibited CD40-induced autophagic killing of *T. gondii* whereas it had no effect on anti-*T. gondii* activity induced by IFN-γ (Figure 2F). We also examined the effects of a neutralizing anti-Tat mAb on the autophagic killing of *T. gondii*. MDM infected with pseudotyped HIV-1 were unable to inhibit anti-*T. gondii* activity in response to CD40 stimulation when an anti-Tat mAb was added to the culture (Figure 2G). Finally, we confirmed this observation in MDM from HIV-1^+^ patients whose cells were refractory to CD40-induced killing of *T. gondii*. In the presence of anti-Tat mAb, MDM from CD40 non-responders exhibited anti-*T. gondii* activity similar to that of MDM from healthy controls (Figure 2H). Taken together, HIV-1 Tat inhibited autophagy.

### HIV-1 impairs autophagy in bystander macrophages/monocytic cells through Src – Akt signaling

**Figure 3:**
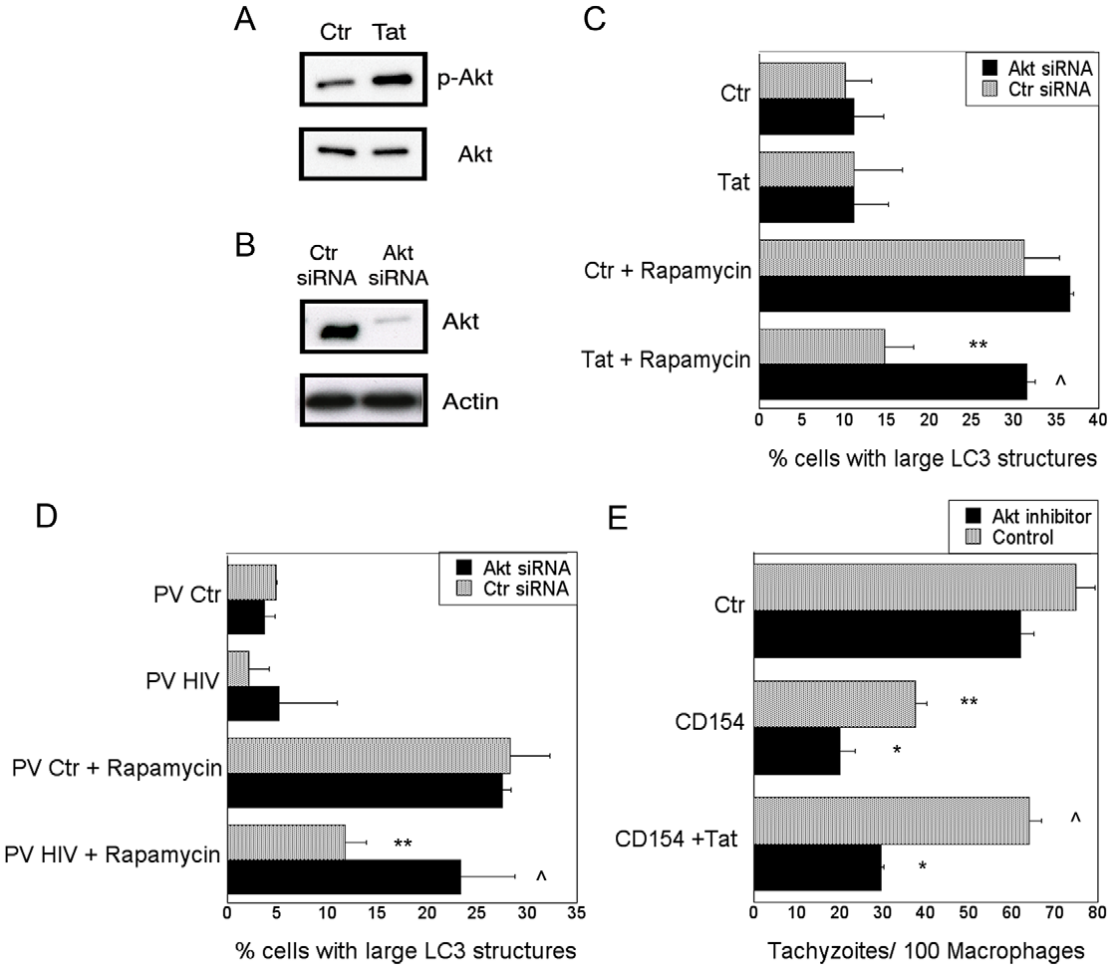
HIV-1 inhibits autophagy in bystander macrophages/monocytic cells through Akt. *A*, MDM incubated with or without HIV-1 Tat (1 ng/ml) were examined for expression of total Akt and phospho-Akt by immunoblot. *B*, MonoMac6 cells were transfected with siRNA against Akt_1_ or control siRNA. Expression of Akt and actin were examined 72 h post-transfection. *C*, MonoMac6 cells transfected with control or siRNA directed against Akt_1_ were transfected with LC3-eGFP. Cells were incubated with or without HIV-1 Tat (100 pg/ml) overnight followed by stimulation with rapamycin. Autophagy was assessed by examining expression of large LC3^+^ structures. *D*, MonoMac6 cells were transfected with control siRNA or siRNA directed against Akt. Cells were transfected with LC3-eGFP and incubated with MonoMac6 cells infected with pseudotyped control virus (PV Ctr) or pseudotyped HIV-1 (PV HIV). Cultures were treated with or without rapamycin. Autophagy was assessed by examining expression of large LC3^+^ structures. *E*, MDM were treated with Akt inhibitor IV (1.25 µM) or vehicle followed by incubation with HIV-1 Tat. Macrophages were incubated with or without CD154, challenged with *T. gondii* and assessed for parasite load at 24 h. Data are representative of 3 independent experiments presented as means ± SEM; *p≤0.05, **p≤0.01, ∧p≥0.05.

HIV-1 can activate Akt [32], [33], which is an inhibitor of autophagy [34], [35]. To determine if Akt mediates the inhibitory effects of Tat on autophagy, we initially examined if Tat induced activation of Akt in macrophages. MDM exhibited enhanced phosphorylation of Akt when incubated with HIV-1 Tat (Figure 3A). Next, we determined if Akt signaling was necessary for the inhibition of autophagy induced by HIV-1 Tat in bystander cells. To this end, we silenced Akt in MonoMac6 cells by utilizing siRNA (Figure 3B). Fluorescent microscopy of LC3-eGFP^+^ MonoMac6 cells revealed that HIV-1 Tat was unable to inhibit autophagy when these cells were transfected with Akt_1_ siRNA (Figure 3C). Additionally, cells infected with pseudotyped HIV-1 were no longer able to inhibit autophagy in MonoMac6 cells that had silenced Akt_1_ (Figure 3D). Similar results were obtained with Akt inhibitor IV (Figure S2A). Incubation with Akt inhibitor IV also ablated the ability of HIV-1 Tat to block CD40-dependent autophagic killing of *T. gondii* (Figure 3E). These results indicate that HIV-1 inhibits autophagy in bystander primary macrophage and monocytic cells through an Akt-dependent pathway.

**Figure 4:**
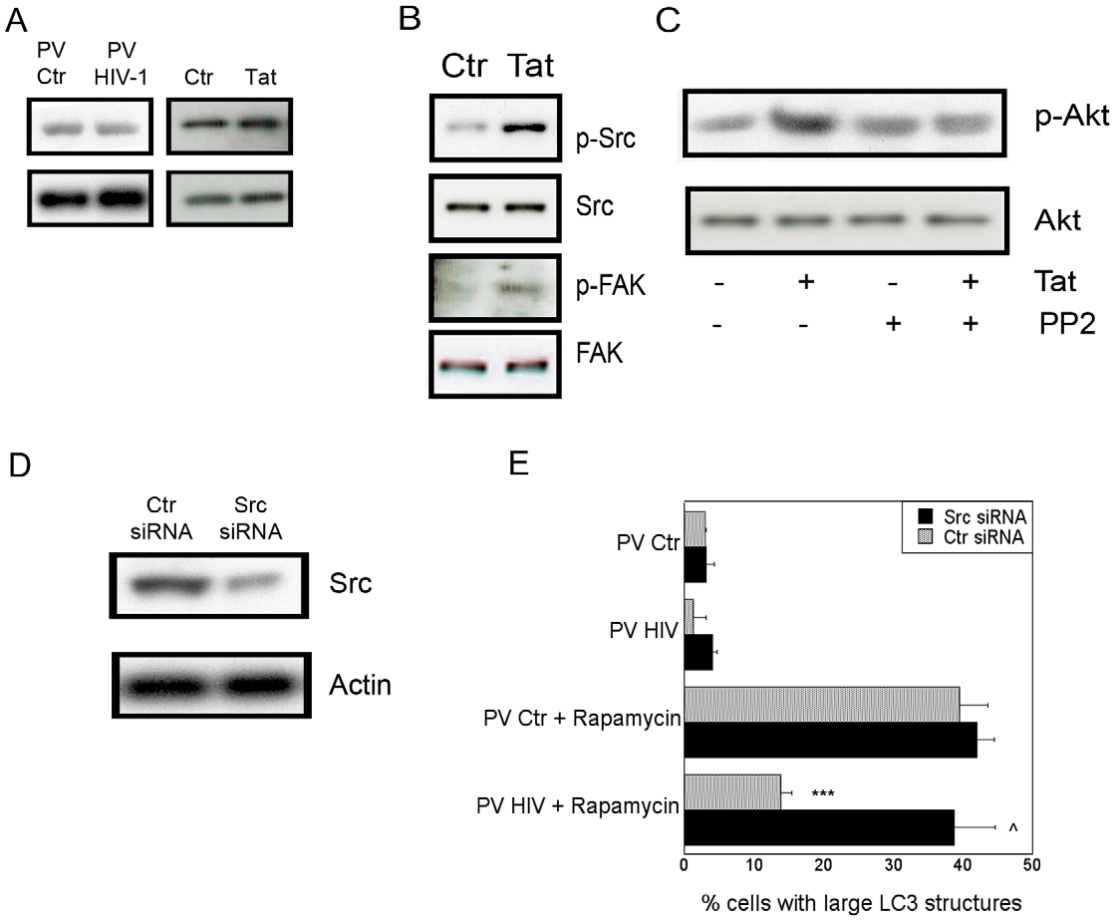
HIV-1 inhibits autophagy in bystander macrophages/monocytic cells through Src and Akt. *A*, MonoMac6 cells were cultured with MonoMac6 cells infected with pseudotyped control virus (PV Ctr) or pseudotyped HIV-1 (PV HIV) or treated with HIV-1 Tat (100 pg/ml). Culture lysates were examined by immunoblot for expression of PTEN and actin. *B*, MDM were incubated with or without HIV-1 Tat and examined for expression of Src, phospho-Src, FAK and phospho-FAK by immunoblot. *C*, MDM were incubated with PP2 (20 µM) or vehicle followed by incubation with HIV-1 Tat. Expression of total Akt and phospho-Akt were examined by immunoblot. *D*, MonoMac6 cells were transfected with control siRNA or siRNA directed against Src and examined for expression of Src and actin. *E*, MonoMac6 cells transfected with control siRNA or siRNA directed against Src were transfected with LC3-eGFP and incubated with pseudotyped control virus (PV Ctr) or pseudotyped HIV-1 (PV HIV)-infected MonoMac6 cells overnight. Cultures were treated with or without rapamycin. Autophagy was assessed by examining expression of large LC3^+^ structures. Data are representative of 3 independent experiments presented as means ± SEM; ***p≤0.001, ∧p≥0.05.

To further investigate the manner in which HIV-1 modulates autophagy in non-infected cells we examined signaling molecules upstream of Akt. Phosphatase and tensin homologue (PTEN) is a potent inhibitor of Akt signaling and it has been reported that HIV-1 can reduce levels of PTEN [32]. However, we did not detect reduction of PTEN expression in cultures of pseudotyped HIV-1 infected cells or after macrophage treatment with HIV-1 Tat (Figure 4A). We then examined the phosphorylation state of Src and FAK, classical activating kinases upstream of Akt [36]. MDM treated with HIV-1 Tat exhibited increased phosphorylation of Src and FAK (Figure 4B). Moreover, treatment of macrophages with the Src inhibitor PP2 prior to addition of HIV-1 Tat blocked Akt phosphorylation (Figure 4C), indicating that activation of Src leads to Akt phosphorylation. Therefore, we examined the role of Src in HIV-1-induced inhibition of autophagy by utilizing siRNA (Figure 4D). Similar to the studies with Akt, cells infected with pseudotyped HIV-1 were unable to inhibit autophagy in MonoMac6 cells that had Src silenced by siRNA (Figure 4E). These results were also reproduced in MonoMac6 cells exposed to HIV-1 Tat (Figure S2B). Taken together, these studies indicate that Src and Akt are required for HIV-1-induced inhibition of autophagy in non-infected primary macrophage and monocytic cells.

### CXCR4, VEGFR1 and β-integrins are required for inhibition of autophagy caused by HIV-1

**Figure 5:**
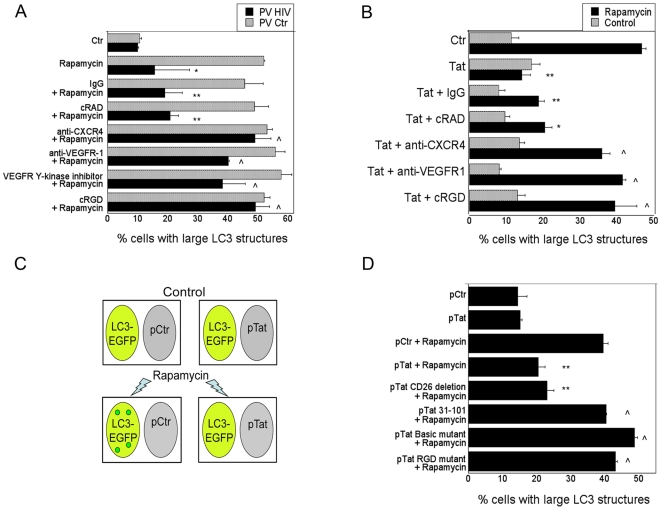
HIV-1 inhibits autophagy in bystander macrophages/monocytic cells through CXCR4, VEGFR and β-integrin. *A*, MonoMa6 cells were transfected with LC3-eGFP and incubated with cells infected with pseudotyped control virus (PV Ctr) or pseudotyped HIV-1 (PV HIV). Cultures were treated overnight with control mAb, control peptide cRAD, anti-CXCR4 mAb, anti-VEGFR1 mAb, VEGFR1 tyrosine kinase inhibitor, or β-integrin blocking peptide cRGD. MonoMac6 cells were treated with or with out rapamycin and assessed for large LC3^+^ structures. *B*, MonoMac6 cells transfected with LC3-eGFP were treated overnight with or without Tat (100 pg/ml) and either control mAb, control peptide cRAD, anti-CXCR4 mAb, anti-VEGFR1 mAb or β-integrin blocking peptide, cRGD. MonoMac6 cells were treated with or without rapamycin and assessed for large LC3^+^ structures. *C*, Schematic representation of MonoMac6 cells transfected with LC3-eGFP cultured overnight with MonoMac6 cells transfected with a control plasmid or a plasmid encoding Tat. Cultures were treated with rapamycin and assessed for large LC3^+^ structures. *D*, MonoMac6 cells transfected with LC3-eGFP were cultured overnight with MonoMac6 cells transfected with a control plasmid or a plasmid encoding either wild-type Tat, Tat CD26 mutant, Tat chemokine-like domain mutant (Tat_31–101_), Tat basic domain mutant, or Tat RGD mutant as depicted in panel (C). Cultures were treated with rapamycin and assessed for large LC3^+^ structures. Data are representative of 4 independent experiments presented as means ± SEM; *p≤0.05, **p≤0.01, ∧p≥0.05.

Since HIV-1 Tat increased phosphorylation of Src and FAK in macrophages, we examined the role of receptors known to signal through these molecules: chemokine receptors, VEGFR and β-integrin [37], [38], [39]. To this end, we blocked engagement of these surface molecules on LC3-eGFP^+^ MonoMac6 cells prior to incubation with pseudotyped HIV-1 infected cells and treatment with rapamycin. A blocking mAb against CXCR4 was used because CXCR4 is a predominant chemokine receptor on macrophages [40] and treatment of MonoMac6 cells with recombinant SDF, the ligand for CXCR4, caused inhibition of autophagy (Figure S3). VEGFR signaling was inhibited with a mAb against VEGFR1 (the predominant form of VEGFR on macrophages), or a VEGFR1 tyrosine-kinase inhibitor. β-integrin signaling was inhibited with a blocking RGD peptide. Neutralization of any of these molecules impaired the ability of cells infected with pseudotyped HIV-1 to inhibit autophagy in bystander cells (Figure 5A). Similar results were obtained with HIV-1 Tat (Figure 5B). These results suggest that all three cell surface molecules are required for successful inhibition of autophagy by HIV-1.

HIV-1 Tat has been reported to interact with chemokine receptors, VEGFR, β-integrins and CD26 through the chemokine-like domain, basic domain, RGD domain and CD26 binding domain respectively [24], [25], [41], [42], [43]. To further confirm the role of cell surface molecules in the inhibition of autophagy, we assessed the ability of Tat with mutations in the above domains to block rapamycin induced autophagy. To this end, we cultured LC3-eGFP^+^ MonoMac6 cells with cells transfected with a control plasmid, a plasmid that encoded wild type HIV-1 Tat (Figure 5C), or HIV-1 Tat with mutations at the CD26-binding, chemokine-like (Tat_31–101_), basic or RGD domains. Equivalent expression of each protein was corroborated by immunoblot using an anti-HA antibody (not shown). Cells that expressed wild type HIV-1 Tat inhibited autophagy in LC3-eGFP^+^ MonoMac6 cells (Figure 5D). Similar results were obtained with cells that expressed HIV-1 Tat with deletion of the CD26 binding domain. In contrast, cells that expressed HIV-1 Tat with mutations at the chemokine-like, basic, or RGD domains lacked the ability to significantly inhibit autophagy (Figure 5D). These results did not correlate with the transactivation activity of HIV-1 Tat since wild type HIV-1 Tat, and the RGD domain mutant exhibited transactivation activity while the remaining mutants were devoid of this activity (not shown). Taken together, these results indicate that HIV-1 inhibits autophagy in non-infected cells in a manner dependent on chemokine, VEGFR and β-integrin signaling. Importantly, inhibition of autophagy in bystander monocytic cells/macrophages caused by HIV-1-infected cells and HIV-1 Tat exhibit similar features since both are dependent on Src-Akt, as well as CXCR4, VEGFR and β-integrin.

### IL-10 and STAT3 are required for inhibition of autophagy induced by HIV-1

**Figure 6:**
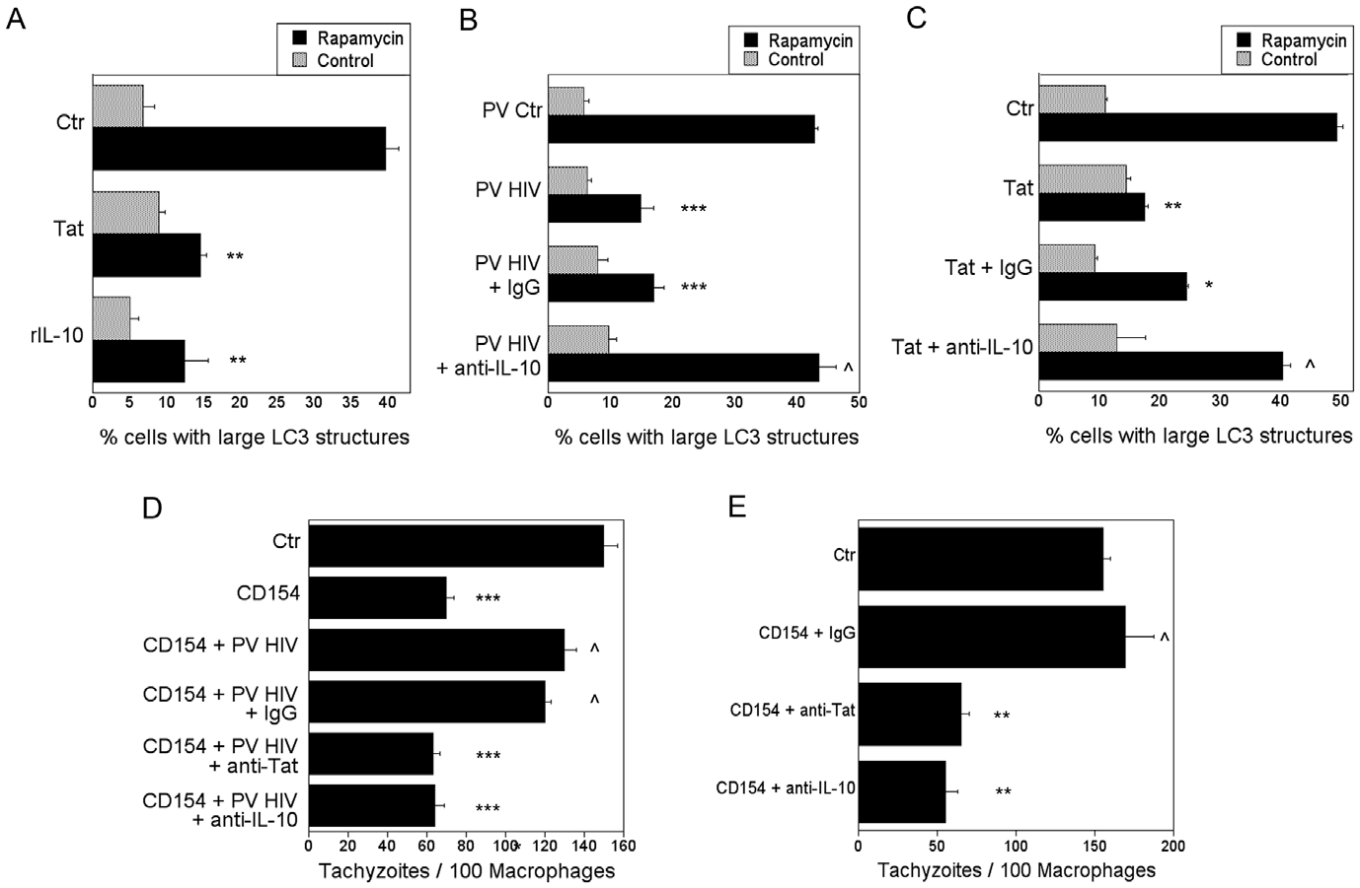
IL-10 is required for HIV-1 to inhibit autophagy in bystander macrophages/monocytic cells. *A*, MonoMac6 cells transfected with LC3-eGFP were incubated with HIV-1 Tat (100 pg/ml) or IL-10 (100 pg/ml) overnight. Cells were then stimulated with rapamycin. Autophagy was assessed by examining expression of large LC3^+^ structures. *B*, MonoMac6 cells transfected with LC3-eGFP were incubated with MonoMac6 cells infected with pseudotyped control virus (PV Ctr) or pseudotyped HIV-1 (PV HIV). Cultures were treated with either neutralizing anti-IL-10 or control mAb overnight. Cells were then stimulated with rapamycin. Autophagy was assessed by examining expression of large LC3^+^ structures. *C*, MonoMac6 cells transfected with LC3-eGFP were incubated with HIV-1 Tat (100 pg/ml) plus either neutralizing anti-IL-10 or control mAb overnight. Cells were then stimulated with rapamycin. Autophagy was assessed by examining expression of large LC3^+^ structures. *D*, MDM infected with pseudotyped HIV-1 were incubated with uninfected MDM. Cells were cultured in the presence of either neutralizing anti-Tat, neutralizing anti-IL-10 or control mAb. After stimulation with CD154, monolayers were challenged with *T. gondii*. The number of parasite per 100 macrophages was examined at 24 h by light microscopy. *E*, MDM taken from a representative CD40 non-responder HIV-1^+^ infected patient were cultured in the presence of neutralizing anti-Tat, anti-IL-10 or control mAb. After stimulation with CD154, monolayers were challenged with *T. gondii*. The number of parasite per 100 macrophages was examined at 24 h by light microscopy. Data are representative of 3 independent experiments presented as means ± SEM; *p≤0.05, **p≤0.01, ***p≤0.001, ∧p≥0.05.

Our data indicate that the presence of low percentages of cells infected with HIV-1 or low concentrations of HIV-1 Tat were sufficient to launch a robust inhibition of autophagy in bystander macrophages/monocytic cells. We explored if HIV-1 utilizes an additional pathway leading to an effective inhibition of autophagy. HIV-1 and HIV-1 Tat upregulate IL-10 [44], [45], [46], [47], [48]. Indeed, we observed that MDM exhibited increased secretion of IL-10 after exposure to 100 pg/ml of HIV-1 Tat (data not shown). Thus, we explored IL-10 signaling as a possible additional mechanism by which HIV-1 impairs autophagy. Treatment of MonoMac6 cells with 100 pg/ml recombinant human IL-10 impaired the induction of autophagy in response to rapamycin (Figure 6A). Interestingly, however, the concentration of IL-10 (30 pg/ml) detected after HIV-1 Tat treatment was insufficient for inhibition of autophagy alone (data not shown). Therefore, we determined whether cells infected with pseudotyped HIV-1 or HIV-1 Tat require the presence of IL-10 to inhibit autophagy. In the presence of a neutralizing anti-IL-10 mAb, MonoMac6 cells infected with pseudotyped HIV-1 no longer inhibited autophagy in rapamycin-treated non-infected MonoMac6 cells (Figure 6B). Similarly, HIV-1 Tat was unable to impair autophagy in rapamycin-treated MonoMac6 cells incubated with anti-IL-10 mAb (Figure 6C).

Furthermore, IL-10 was also required for HIV-1-induced inhibition of autophagic killing of *T. gondii*. MDM infected with pseudotyped HIV-1 did not inhibit anti-*T. gondii* activity in response to CD40 stimulation when an anti-IL-10 mAb was present (Figure 6D). Finally, in the presence of anti-IL-10 mAb, MDM from HIV-1^+^ patients whose MDM were refractory to CD40-induced autophagic killing of *T. gondii*, exhibited anti-*T. gondii* activity similar to that of MDM from healthy controls (Figure 6E). Taken together, HIV-1 requires IL-10 to inhibit autophagy in bystander macrophages/monocytic cells.

**Figure 7:**
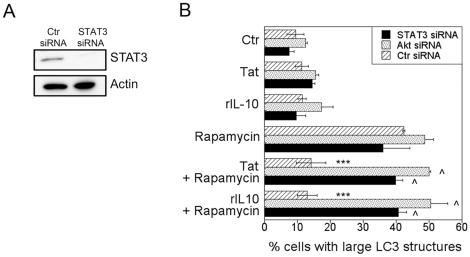
STAT3 and Akt are required for HIV-1 and IL-10 to inhibit autophagy. *A*, MonoMac6 cells were transfected with siRNA against STAT3 or control siRNA. Expression of STAT3 and actin were examined 72 h post-transfection. *B*, MonoMac6 cells transfected with control siRNA or siRNA directed against STAT3 or Akt were transfected with LC3-eGFP. Cells were incubated with or without HIV-1 Tat or IL-10 (both at 100 pg/ml) overnight followed by stimulation with rapamycin. Autophagy was assessed by examining expression of large LC3^+^ structures. Data are representative of 3 independent experiments presented as means ± SEM; ***p≤0.001, ∧p≥0.05.

**Figure 8:**
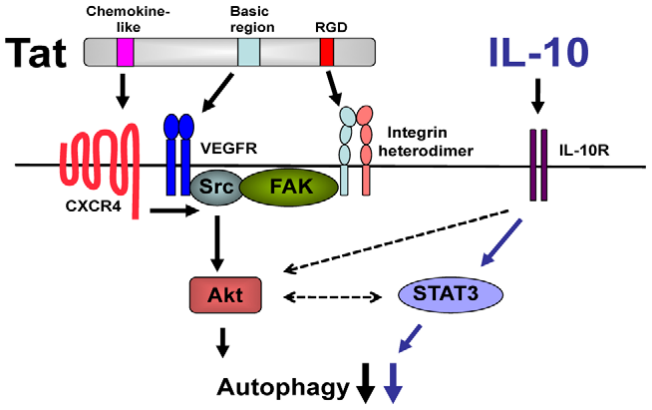
A schematic representation of the mechanism of HIV-1-induced inhibition of autophagy. HIV-1 Tat interaction with the cell surface receptors CXCR4, VEGFR, and β-Integrin induces signaling through Src, it's binding partner FAK, and Akt. Concomitant upregulation of IL-10 by HIV-1 results in STAT3 activity, which functions in cooperation with Akt to initiate a robust inhibition of autophagy.

STAT3 is a major signaling molecule downstream of IL-10 [49], [50]. Thus, we examined the role of STAT3 in the inhibition of autophagy induced by HIV-1 and IL-10. Transfection of MonoMac6 cells with siRNA directed against STAT3 reduced expression of this protein (Figure 7A). Silencing of STAT3 prevented IL-10 and HIV-1 Tat from inhibiting autophagy induced by rapamycin (Figure 7B). The PI3K/Akt pathway has also been reported to mediate IL-10 signaling [51], [52], [53]. Indeed, silencing of Akt prevented IL-10 from inhibiting autophagy induced by rapamycin (Figure 7B). Therefore, IL-10 signaling through STAT3 and Akt is necessary for blockade of autophagy. IL-10 appears unlikely to be the sole mediator of inhibition of autophagy. In this regard, the concentration of IL-10 (30 pg/ml) detected in MDM cultures after HIV-1 Tat treatment was insufficient for blockade of rapamycin-induced autophagy (data not shown). Moreover, IL-10 production induced by HIV-1 Tat is reported to be independent of the basic and RGD domains while these sites are required for inhibition of autophagy [54], [55]. Taken together, these results support a model whereby HIV-1 and IL-10 act in cooperation through Akt and STAT3 to impair autophagy in bystander macrophage/monocytic cells (Figure 8).

## Discussion

Microbial virulence factors have been reported to inhibit autophagy by binding to Atg proteins [6], [8], [17]. Here we report that a pathogen impairs autophagy by acting at upstream events in the regulation of autophagy. HIV-1 inhibits autophagy in bystander non-infected macrophages/monocytic cells. This inhibition is dependent on Akt, its upstream activator Src as well as on STAT3 and IL-10. The simultaneous signaling by Akt and STAT3 contribute to the robust inhibition of autophagy induced by HIV-1. We believe that data from this study provide a novel insight into the strategies used by a pathogen to inhibit autophagy.

HIV-1 can affect a variety of signaling cascades. In this regard, HIV-1 induces activation of Akt, Src and FAK [25], [32], [33], [56]. Our studies using gene knockdown and pharmacological inhibition of Src and Akt identified them as mediators of HIV-1-induced inhibition of autophagy in bystander macrophages/monocytic cells. Moreover, β-integrins, chemokine receptors and growth factor receptors such as VEGFR, are well-described stimulators of Akt [37], [38], [39]. Indeed, blockade of any of these surface molecules impairs the ability of HIV-1 to inhibit autophagy in bystander macrophage/monocytic cells. These findings suggest that signaling through these receptors synergize to trigger inhibition of autophagy.

HIV-1 infection results in dysregulation of IL-10. Cells exposed to various HIV-1 proteins, including Tat, exhibit increased IL-10 production; furthermore, HIV-1^+^ patients have increased serum levels of IL-10 [44], [45], [47], [48]. We identified IL-10 as an inhibitor of autophagy and we report that HIV-1 also acts through IL-10 to impair autophagy in bystander macrophage/monocytic cells. The role of IL-10 is further supported by silencing of STAT3, a major signaling molecule downstream of this cytokine. Relevant to these findings is the report that telomere 3′ overhang-specific DNA oligonucleotides appear to induce autophagy by inhibiting STAT3 [57]. Thus, our studies indicate that inhibition of autophagy is part of the constellation of inhibitory effects of IL-10 on macrophage effector functions. IL-4 and IL-13 are cytokines that inhibit autophagy through either Akt or STAT6 [58]. We report that IL-10 impairs autophagy not only through STAT3 but also via Akt. While our results support the existence of at least two pathways by which HIV-1 inhibits autophagy in bystander macrophages, Src – Akt and STAT3, the possibility of crosstalk between signaling cascades cannot be ruled out. For example, there is evidence for crosstalk and cooperation between STAT3 and Akt [59], [60]. Additionally, not only IL-10 but also CXCR4 activates STAT3 [61]. Regardless of potential additional interactions between pathways, our studies revealed that signaling by Akt and STAT3 are required for HIV-1-induced inhibition of autophagy in bystander macrophages/monocytic cells.

Our work reveals that HIV-1-infected cells inhibit autophagy in non-infected macrophages/monocytic cells. Various HIV-1 molecules affect the function of cells not infected with the virus. Our studies with pseudotyped HIV-1 indicate that autophagy is inhibited in bystander macrophages/monocytic cells despite the lack of expression of Env and Nef. Moreover, blockade of VEGFR, CXCR4 and β-integrin signaling restored autophagy. This raised the possibility that HIV-1 Tat inhibits autophagy since this molecule has sites with homology to natural ligands for those receptors and HIV-1 Tat has been reported to exhibit pleiotropic effects on uninfected bystander cells through interaction of HIV-1 Tat domains with VEGFR, chemokine receptors and β-integrins [24], [25], [26], [27], [28], [29]. However, the difficulty in measuring extracellular HIV-1 Tat owing to the lack of suitable commercially available reagents and the high concentrations of HIV-1 Tat used in some studies have created controversy in the field. We report that low concentrations of HIV-1 Tat inhibit autophagy in macrophages/monocytic cells. The role of HIV-1 Tat as an inhibitor of autophagy is further supported by the following observations: i) Cells transfected with a *tat*-encoding plasmid inhibit autophagy in bystander monocytic cells, ii) A neutralizing anti-Tat mAb restores rapamycin-induced autophagy and CD40-dependent autophagic killing of *T. gondii* in macrophages/monocytic cells exposed to HIV-1-infected cells, iii) Neutralization of HIV-1 Tat restores autophagic killing of *T. gondii* in macrophages from HIV-1^+^ patients. Importantly, the effects of HIV-1 Tat on autophagy emulate the inhibition of autophagy in bystander monocytic cells/macrophages caused by cells infected with HIV-1, including reliance on host membrane receptors, Akt, Src, and IL-10 signaling.

The effective inhibition of autophagy is likely explained by cooperation between HIV-1 and upregulated IL-10. Modulation of IL-10 by exogeneous HIV-1 Tat is reliant on amino acid residues 1–45 of HIV-1 Tat, which includes the chemokine-like domain [54], [55], however, not only the chemokine-like domain but also the basic and RGD domain of HIV-1 Tat are required for inhibition of autophagy. This is supported not only by studies using inhibitors of VEGFR, β-integrin and CXCR4 signaling but also by studies in which monocytic cells are exposed to cells that express either wt *tat* or *tat* with mutations in the domains that interact with these receptors. The synergistic signaling events induced by Tat along with the presence of IL-10 provide an effective inhibition of autophagy. Consistent with the notion that IL-10 alone is insufficient for the inhibition of autophagy prompted by HIV-1, IL-10 by itself does not block autophagy at the concentrations detected in MDM cultures incubated with HIV-1 Tat. Our results of IL-10 inhibition of autophagy support the cooperation of Tat signaling. Therefore, HIV-1 and inducible IL-10 likely function in combination to successfully inhibit autophagy.

There is increasing evidence of the ability of HIV-1 to manipulate autophagy. Env has been reported to induce autophagy in bystander CD4^+^ T cells through the fusogenic effect of gp41 [62]. In contrast, Env was unable to induce autophagy in bystander macrophages/monocytic cells [18]. HIV-1 plays a complex role in modulation of autophagy within cells infected by the virus. Autophagy is inhibited in CD4^+^ T cells acutely infected with HIV-1 [18], [63]. In macrophages/monocytic cells infected with HIV-1, while the early stages of autophagy are stimulated, the degradative stage of this process is inhibited by Nef [17], [18]. Although those studies provided an important insight into modulation of autophagy within HIV-1-infected cells, the fact that only a fraction of cells *in vivo* are estimated to be infected stresses the importance of determining whether HIV-1 inhibits autophagy in bystander cells. Little is known in this regard except for the report that autophagy is inhibited in bystander neurons treated with supernatants from microglia obtained from SIV-1 infected monkeys [19]. However, it was not determined if the effect was a direct consequence of SIV-1. Indeed, it was postulated that the bystander effect might be due to excitotoxic and pro-inflammatory products secreted by microglia. We on the other hand, demonstrate that HIV-1 is able to induce a robust inhibition of autophagy in bystander macrophages/monocytic cells. This phenomena may explain why macrophages from a subset of HIV-1^+^ patients exhibit defective CD40-dependent autophagic killing of *T. gondii*. In this regard, macrophages from two CD40-non-responder HIV-1^+^ patients tested appeared to have detectable cells with productive HIV-1 infection since approximately 0.5% of the macrophages were p24^+^ by flow cytometry (Andrade and Subauste, unpublished observations). This supports that a small proportion of HIV-1 infected cells can have a significant impact on autophagy in HIV-1 non-infected cells.

Our findings are likely of relevance to various aspects of HIV-1-induced disease. The role of autophagy as an immune effector mechanism for targeted destruction of several intracellular pathogens, including *M. tuberculosis*, *T. gondii*, and *Salmonella typhimurium* [4], [11], [64], [65] suggests that the inhibition of autophagy in bystander macrophages may contribute to the susceptibility of HIV-1^+^ patients to tuberculosis, toxoplasmosis and/or salmonellosis. The fact that autophagy can promote antigen presentation [13], [15] raises the possibility that inhibition of autophagy may promote pathogen immune evasion. Autophagy is critical for control of protein quality and prevention of neurodegenerative diseases [66]. HIV-1 associated dementia (HAD) is becoming a major problem in HIV-1^+^ patients. It has been proposed that dysregulation of autophagy by HIV-1 contributes to HAD [19]. The fact that patients with HAD exhibit perivascular infiltration with HIV-1-infected monocytes [67] and the proposed link between HIV-1 Tat and HAD [68] suggest that the mechanism of autophagy inhibition reported here may contribute to the development of HAD.

Studies in animal models of neurodegenerative disorders revealed that pharmacologic manipulation of autophagy is of therapeutic value [69], [70]. The complex effects of HIV-1 on the autophagy pathway suggest that therapeutic benefit may not be easily achieved by generalized manipulation of autophagy through targeting autophagy proteins. Our studies raise the possibility of a more selective approach to manipulate autophagy by targeting regulatory pathways. Since blockade of one signaling pathway (for example VEGFR) remarkably diminishes the ability of HIV-1 to impair autophagy in bystander cells, this mechanism may be more easily exploited for therapeutic use in HIV-1^+^ patients. Of note, inhibitors of VEGFR, Src or Akt signaling are being explored for cancer treatment [71], [72], [73].

In summary, we report that a pathogen inhibits autophagy by modulating counter-regulatory signaling pathways, this inhibitory effect can have global characteristics since it affects bystander (non infected) macrophages/monocytic cells and approaches currently developed for treatment of various disorders in humans prevent inhibition of autophagy. The findings presented herein may be relevant to the development of opportunistic infections, immune evasion and neuro-degeneration in HIV-1^+^ patients. These studies may open up the possibility of novel treatments for complication of HIV-1 infection based on the use of agents currently developed for the management of other disorders in humans.

## Materials and Methods

### Ethics statement

Written informed consent was obtained from all subjects, and the human experimentation guidelines of the US Department of Health and Human Services were followed. The study was approved by the Institutional Review Board of the University of Cincinnati.

### HIV-1^+^ patients and control subjects

Blood samples were collected from 23 HIV-1 infected patients followed at the Infectious Diseases Center of the University of Cincinnati. Plasma viral load was measured by RT-PCR (Amplicor, Roche). Blood samples were collected from 10 healthy volunteers and used as control subjects.

### Macrophage and monocytic cells

Human MDM were obtained as previously described [11], [74], MonoMac6 cells were a gift from Rene de Waal Malefyt (DNAX Research Institute, Palo Alto, CA). Stable expression of LC3- eGFP was achieved in THP-1 cells using a vector provided by Christian Munz (University Hospital of Zurich, Switzerland). In certain experiments, macrophages and monocytic cells were treated with varying concentrations of recombinant Tat_1–101_ (Tecnogen), recombinant human IL-10 (100 pg/ml; PeproTech), recombinant IFN-γ (200 U/ml; PeproTech), neutralizing mAb against either Tat (10 µg/ml; Immunodiagnostics), IL-10 (10 µg/ml; BD Biosciences), TNF-α (10 µg/ml; Endogen), CXCR4 (25 µg/ml; R&D Systems), VEGFR1 (10 µg/ml; R&D Systems), control mouse IgG (BD Biosciences), goat IgG (Jackson ImmunoResearch Laboratories, Inc.), Akt inhibitor IV (1.25 µM; Calbiochem), integrin blocking peptide cRGDfV (3 µM; Bachem) or control peptide cRADfV (3 µM; Bachem).

### *T. gondii* infection

MDM were stimulated with CD154 (3 µg/ml; Amgen) for 72 h in the presence of macrophages infected with pseudotyped HIV-1 (see below), recombinant HIV-1 Tat (60 pg/ml added daily for 3 days unless otherwise stated) and/or Akt inhibitor (1.25 µM). Tachyzoites of the RH strain of *T. gondii* were maintained in human foreskin fibroblasts and were used to infect human MDM [11], [74]. Monolayers of macrophages were examined by light microscopy after 24 h to assess parasite load as described [11], [74], [75]. Changes in the number of *T. gondii* infected cells were not due to differences in cell detachment during processing of samples or HIV-1 presence. In addition, cell densities as determined with an eyepiece grid were similar in all experimental groups.

### Pseudotyped HIV-1 infection

To generate pseudotyped HIV-1, we co-transfected 293T cells with VSV-G, pMDL-g/p RRE and either pNL4-3.HSA.E^−^ (obtained from Dr. Nathaniel Landau through the AIDS Research and Reference Reagent Program) or HIV-1 transfer plasmid pHR' as control. pNL4-3.HSA.E^−^ contains a construct for the murine Heat Stable Antigen (HSA, CD24) fused in frame to the *nef* initiator methionine codon. The *env* gene is not expressed due to a 5′ frameshift. Thus, viral particles formed after initial infection with VSV-G pseudotyped HIV-1 lack HIV-1 gp160 and are unable to infect other cells. pHR' contains viral LTR and does not express any HIV-1 proteins. Supernatants were collected at 24 and 48 h, passed through a 0.45 µm filter, concentrated by ultracentrifugation and stored at −80°C. MDM and MonoMac6 cells (1×10^6^ cells/well in 24-well plates) were infected with pseudotyped HIV-1 as described in the presence of polybrene (8 µg/ml; Sigma) in a final volume of 250 µl for 2 h [76]. In certain experiments zidovudine (10 µM, Sigma Chemical) was added 2 h after incubation with HIV-1. Percentages of infected cells were assessed at 48 h by examining expression of cell surface mouse HSA or intracellular p24 by flow cytometry.

### Assessment of Autophagy

MonoMac6 cells were transfected with LC3-eGFP or double transfected with LC3-eGFP and siRNA for Akt_1_, Src, or STAT3 by Amaxa Nucleofection (Amaxa) per manufacturer's protocol. The percentage of LC3-eGFP transfected MonoMac6 cells was consistent amongst all groups. Cells were plated in an 8-well chamber slide and treated with recombinant HIV-1-Tat (100 pg/ml) for 16 hours, then incubated with rapamycin (1 µM; Calbiochem) for 2 h to stimulate autophagy. At least 200 LC3-eGFP^+^ MonoMac6 cells were counted to examine the presence of large (≥1 µm) LC3^+^ structures using a Leica fluorescent inverted microscope at 400× magnification. In additional experiments, MonoMac6 cells were infected with pseudotyped HIV-1, or transfected with either wild-type Tat_101_ plasmid or mutant plasmids. After 48 h these cells were incubated for 16 h with MonoMac6 cells that had been transfected with LC3-eGFP. Autophagy was assessed as described above 2 h after addition of rapamycin. In certain experiments, cultures were treated with bafilomycin A_1_ (100 nM; Sigma Chemical) for 2 h prior to autophagy assessment. In additional experiments, uninfected H9 T cells or H9/HTLV-III T cells (gift from David Canaday) were incubated for 72 h with THP-1 cells that express LC3-eGFP. Autophagy was assessed as described above 2 h after addition of rapamycin. In addition, immunoblot analysis was utilized to determine autophagy induction by expression of LC3 II. MDM or MonoMac6 cells stimulated with HIV-1 Tat with or without rapamycin and bafilomycin A_1_ were lysed and analyzed by immunoblot, as described below, for expression level of LC3 II.

### Transfection of monocytic cells

MonoMac6 cells were transiently transfected with plasmids encoding LC3-eGFP (gift from T. Yoshimori National Institute for Basic Biology, Okazaki, Japan); Tat_1–101_ or the Tat mutants described below using an Amaxa Nucleofector per manufacturer's instructions [11]. Knockdown of Akt_1_, Src and STAT3 were performed by transfecting MonoMac6 cells with previously described siRNA duplexes [77], [78], [79] (Dharmacon Inc.) using an Amaxa Nucleofector. Control siRNA from Dharmacon Inc. was used as control. After 48 h, MonoMac6 cells were transfected with LC3-eGFP.

### Plasmids

A construct of recombinant hemagglutinin (HA)-tagged wild-type Tat (Tat_1–101_); Tat_31–101_ was subcloned into the plasmid pLINK2 using EcoRV and SpeI enzymes. Constructs that encode mutants of Tat were generated by PCR based mutagenesis. Site directed mutagenesis was carried out with QuickChange XL Site- Directed Mutagenesis Kit (Stratagene) following manufacturers instructions. The following oligonucleotides were used to generate mutants: RGD mutant (Tat_R(78)K,D(80)E_) 5′-CCC GCC TCC CAG TCC AAA GGG GAA CCG ACA GGG CCC-3′; basic mutant (Tat_R(53,54,55,56,58,59)A_) 5′- GGC ATC TCC TAT GGC GCG AAG AAG GCG GCA CAG GCA GCA GCA GCT CCT CAG GAC -3′; CD26 binding domain mutant (Tat_21–101_) 5′-GGG GAA TTC CCC TGG AAG CAT CCA GGA AGT CAG CC-3′. Resulting mutants were confirmed by sequencing (Integrated Biotechnologies).

### Immunoblotting

Primary macrophages or MonoMac6 cells treated with recombinant Tat were lysed and assessed for expression of various signaling proteins. In brief, cytoplasmic protein was separated using Tris-HCL gels (BioRad) and transferred to PVDF membranes for probing overnight with primary antibodies directed against Akt, phospho-Akt, Src, phospho-Src, PTEN (all from Cell Signaling Technologies), FAK (Santa Cruz Biotechonologies), phospho-FAK (BD Bioscience), LC3 (MBL Technologies) and HA (Cell Signaling Technologies). The membranes were incubated with corresponding secondary antibodies and the signal was visualized by using a chemilluminescent kit (Pierce Bioscience). In addition, MonoMac6 cells were lysed 72 h after transfection with siRNA for Akt_1_, Src, or STAT3 and probed with antibodies against Akt, Src, STAT3 (R & D Systems) and Actin (Santa Cruz Biotechnologies) to ensure proper knockdown.

### Flow cytometry

Human MDM and MonoMac6 cells were incubated with human IgG (20 µg/ml) for 15 min followed by incubation with anti-CD40, anti-HSA or isotype control mAb (Pharmingen). Cells were fixed with 1% paraformaldehyde and analyzed using an LSR II (BD Biosciences). For detection of intracellular p24, cells were fixed and permeabilized using IntraPrep permeabilization reagent (Beckman Coulter). Thereafter, cells were stained with anti-p24 or isotype control mAb (Beckman Coulter). After fixation with 1% paraformaldehyde, cells were analyzed using an LSRII.

### Statistics

Statistical significance was assessed by 2-tailed Student's *t* test and ANOVA. Values less than 0.05 were considered statistically significant. Linear-regression analysis was performed to determine the relationship between CD40-induced anti-*T. gondii* activity, CD4 counts and viral load.

## Supporting Information

Figure S1 HIV-1 inhibits autophagy in bystander cells. A, MonoMac6 cells infected with pseudotyped HIV-1 (PV HIV) or pseudotyped control virus (PV Ctr) were incubated with uninfected MonoMac6 cells transfected with LC3-eGFP. Cells were treated with or without rapamycin (1 µM) in the presence of bafilomycin A1 (BafA1; 100 nM) and assessed for autophagy by expression of large LC3+ structures. B, THP-1 cells that express LC3-eGFP were incubated for 72 h with either HIV-1-infected or uninfected H9 T cells. Cultures were treated with rapamycin. Autophagy was assessed by examining expression of large LC3+ structures. Data are representative of 2 independent experiments presented as means + SEM; **p<0.01, ***p<0.001, ∧p>0.05.
(0.36 MB TIF)

Figure S2 HIV-1 Tat inhibits autophagy in an Akt-Src- dependent manner. A, MonoMac6 cells transfected with LC3-eGFP were incubated overnight with or with out HIV-1 Tat (100 pg/ml) in the presence of Akt inhibitor (1.25 µM). Cells were then stimulated with rapamycin. Autophagy was assessed by examining expression of large LC3+ structures. B, MonoMac6 cells transfected with control siRNA or siRNA directed against Src were transfected with LC3-eGFP and treated with HIV-1 Tat overnight. Cultures were treated with or without rapamycin. Autophagy was assessed by examining expression of large LC3+ structures. Data are representative of 3 independent experiments presented as means + SEM; **p<0.01, ∧p>0.05.
(0.33 MB TIF)

Figure S3 CXCR4 activation inhibits autophagy. MonoMac6 cells transfected with LC3-eGFP were incubated overnight with SDF-1 (100 pg/ml). Cells were then stimulated with or with out rapamycin. Autophagy was assessed by examining expression of large LC3+ structures. Data are representative of 3 independent experiments presented as means + SEM; **p<0.01.
(0.22 MB TIF)
