# Supplementary figures and images for: HIV-1 Inhibits Autophagy in Bystander Macrophage/Monocytic Cells through Src-Akt and STAT3
Source: PLoS One. 2010 Jul 22;5(7):e11733. doi: 10.1371/journal.pone.0011733 (PMC2908694; doi:10.1371/journal.pone.0011733)

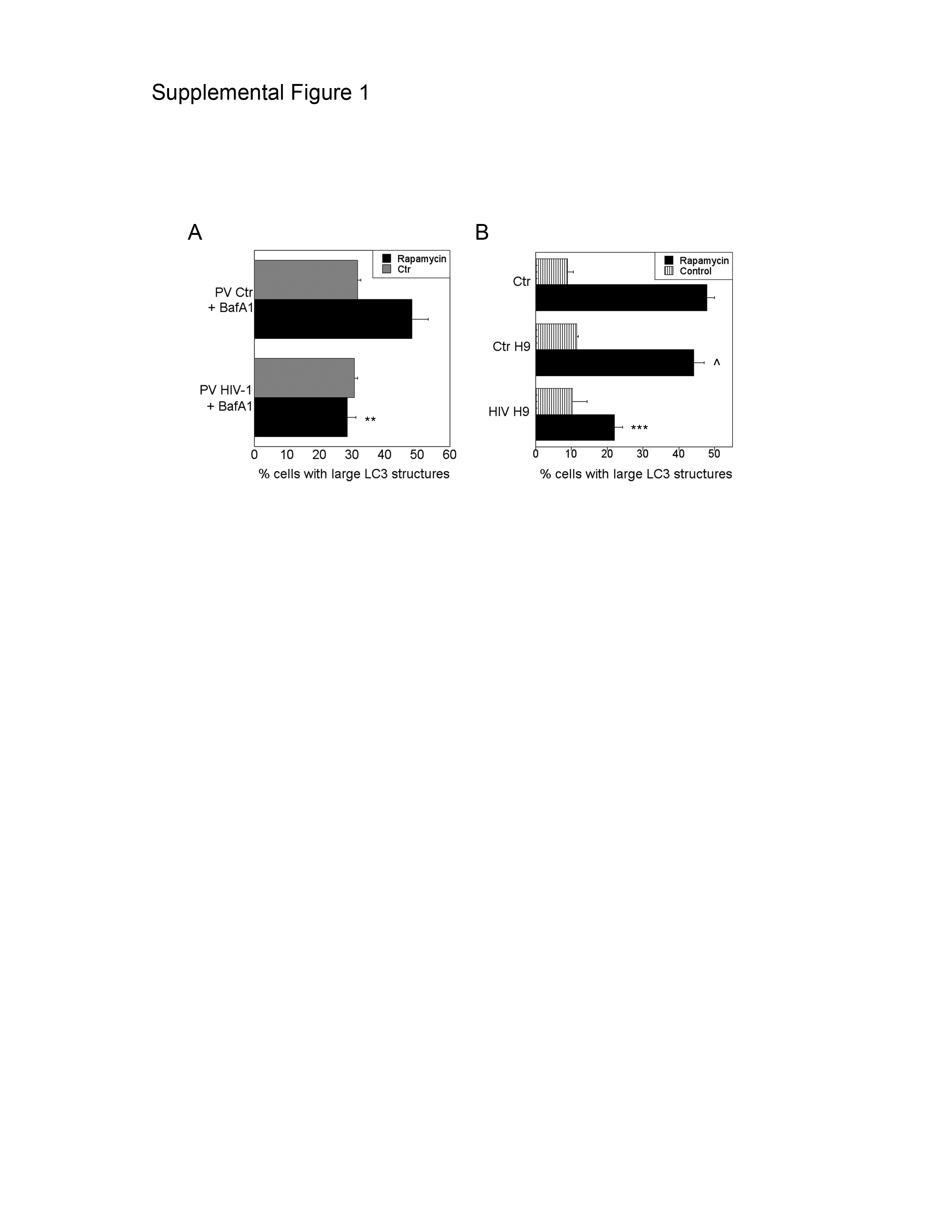

Supplement: Figure S1 — HIV-1 inhibits autophagy in bystander cells. A, MonoMac6 cells infected with pseudotyped HIV-1 (PV HIV) or pseudotyped control virus (PV Ctr) were incubated with uninfected MonoMac6 cells transfected with LC3-eGFP. Cells were treated with or without rapamycin (1 µM) in the presence of bafilomycin A1 (BafA1; 100 nM) and assessed for autophagy by expression of large LC3+ structures. B, THP-1 cells that express LC3-eGFP were incubated for 72 h with either HIV-1-infected or uninfected H9 T cells. Cultures were treated with rapamycin. Autophagy was assessed by examining expression of large LC3+ structures. Data are representative of 2 independent experiments presented as means + SEM; **p<0.01, ***p<0.001, ∧p>0.05. (0.36 MB TIF) [file pone.0011733.s001.tif]

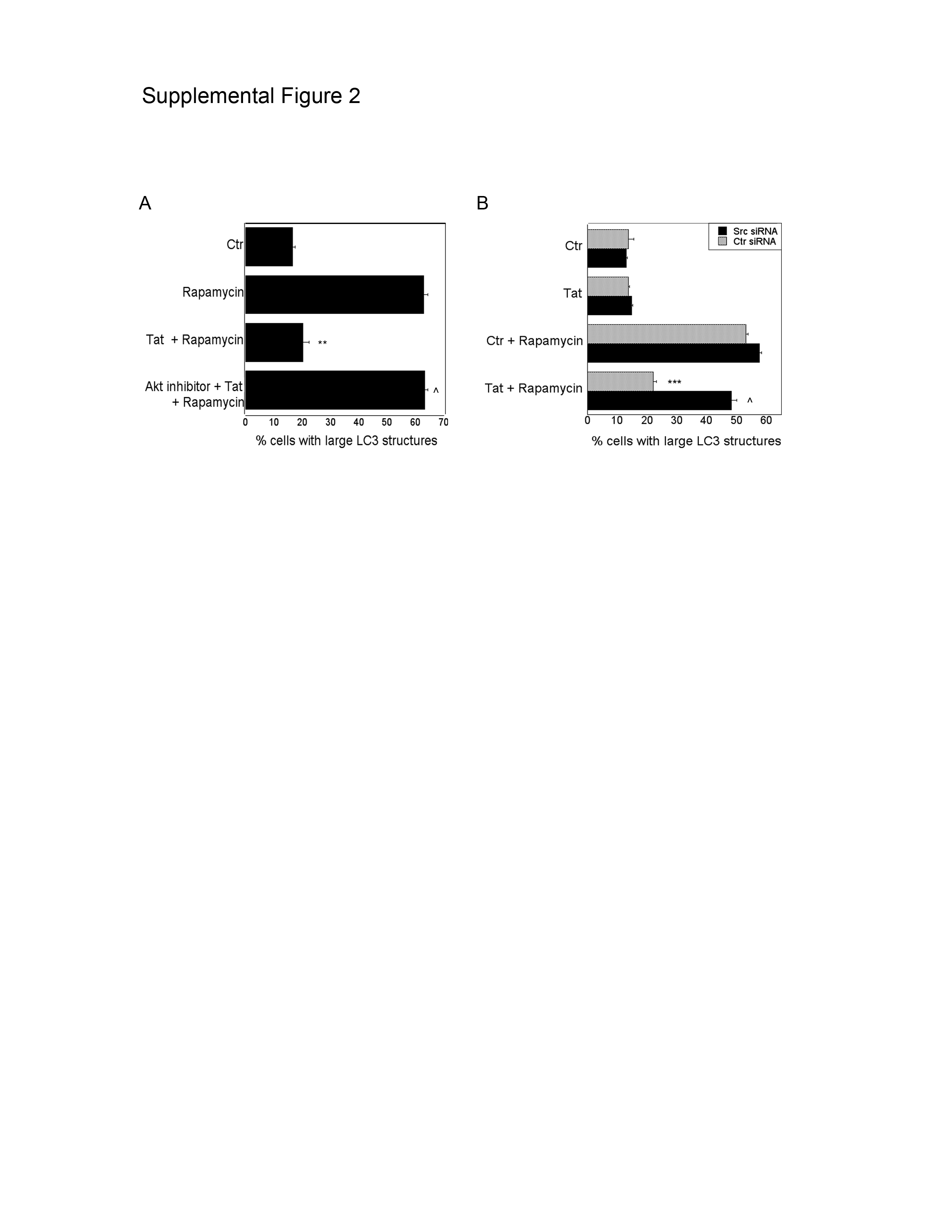

Supplement: Figure S2 — HIV-1 Tat inhibits autophagy in an Akt-Src- dependent manner. A, MonoMac6 cells transfected with LC3-eGFP were incubated overnight with or with out HIV-1 Tat (100 pg/ml) in the presence of Akt inhibitor (1.25 µM). Cells were then stimulated with rapamycin. Autophagy was assessed by examining expression of large LC3+ structures. B, MonoMac6 cells transfected with control siRNA or siRNA directed against Src were transfected with LC3-eGFP and treated with HIV-1 Tat overnight. Cultures were treated with or without rapamycin. Autophagy was assessed by examining expression of large LC3+ structures. Data are representative of 3 independent experiments presented as means + SEM; **p<0.01, ∧p>0.05. (0.33 MB TIF) [file pone.0011733.s002.tif]

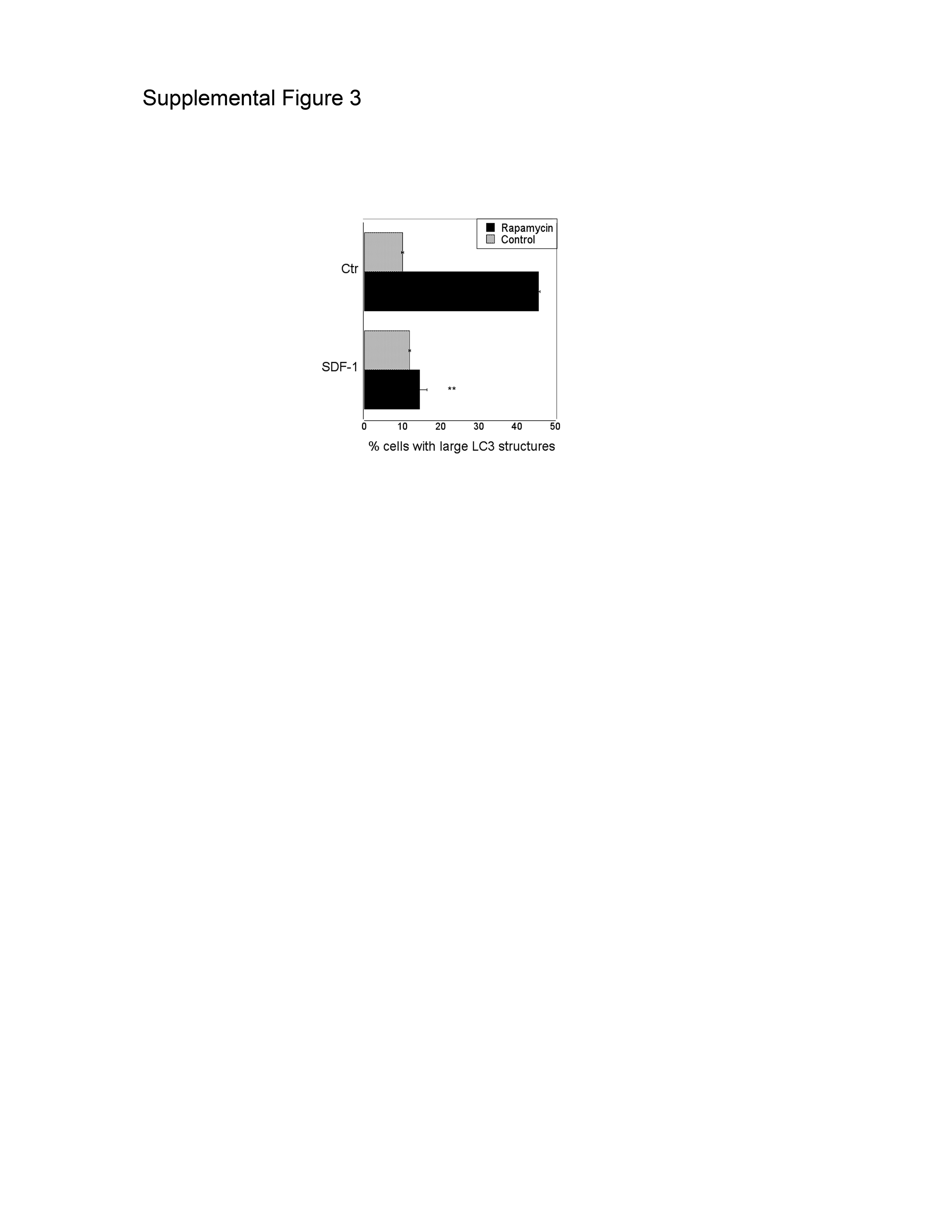

Supplement: Figure S3 — CXCR4 activation inhibits autophagy. MonoMac6 cells transfected with LC3-eGFP were incubated overnight with SDF-1 (100 pg/ml). Cells were then stimulated with or with out rapamycin. Autophagy was assessed by examining expression of large LC3+ structures. Data are representative of 3 independent experiments presented as means + SEM; **p<0.01. (0.22 MB TIF) [file pone.0011733.s003.tif]
